# The Spontaneous Escape Behavior of Silver from Graphite-like Carbon Coatings and Its Effect on Corrosion Resistance

**DOI:** 10.3390/ma16113909

**Published:** 2023-05-23

**Authors:** Deye Li, Wenqiang Wang, Cancan Liu, Luis Alberto Angurel, Germán F. de la Fuente, Bailing Jiang

**Affiliations:** 1 College of Materials Science and Engineering, Nanjing Tech University, Nanjing 211800, China; 202061203169@njtech.edu.cn (D.L.); 202161203202@njtech.edu.cn (W.W.); jiangbail@vip.163.com (B.J.); 2 Instituto de Nanociencia y Materiales de Aragón (CSIC-Universidad de Zaragoza), 50018 Zaragoza, Spain; angurel@unizar.es (L.A.A.); german.delafuente.leis@csic.es (G.F.d.l.F.)

**Keywords:** graphite-like carbon coating, spontaneous escape, silver particles, deposition temperature

## Abstract

Silver-doped graphite-like carbon (Ag-GLC) coatings were prepared on the surface of aluminum alloy and single-crystal silicon by magnetron sputtering under different deposition parameters. The effects of silver target current and deposition temperature, as well as of the addition of CH_4_ gas flow, on the spontaneous escape behavior of silver from the GLC coatings were investigated. Furthermore, the corrosion resistance of the Ag-GLC coatings were evaluated. The results showed that the spontaneous escape phenomenon of silver could take place at the GLC coating, regardless of preparation condition. These three preparation factors all had an influence on the size, number and distribution of the escaped silver particles. However, in contrast with the silver target current and the addition of CH_4_ gas flow, only the change in deposition temperature had a significant positive effect on the corrosion resistance of the Ag-GLC coatings. The Ag-GLC coating showed the best corrosion resistance when the deposition temperature was 500 °C, which was due to the fact that increasing the deposition temperature effectively reduced the number of silver particles escaping from the Ag-GLC coating.

## 1. Introduction

There is a development tendency that the traditional graphite bipolar plates are being replaced by the thinner metallic ones, particularly, in the field of proton exchange membrane fuel cells (PEMFC) [1,2]. Compared with the traditional graphite material, the metallic bipolar plate has many advantages such as a light weight, perfect electrical and thermal conductivity, good gas resistance, high strength and easy processing [3,4]. However, the metallic bipolar plate is easily passivated, which increases the contact resistance. Thus, the design of the functional coatings for these metallic bipolar plates (such as 316L, aluminum alloys, etc.) is a key challenge which needs to be solved in order to enhance their electrical conductivity and to improve their corrosion resistance [5,6]. Graphite-like carbon (GLC) coatings are being considered as ideal functional coatings for increasing their service life [7,8,9]. Usually, GLC coatings contain a certain amount of *sp*^3^ hybrid bonds that reduce their electrical conductivity. For this reason, several studies have explored the possibility of doping the coating with metallic elements, such as Cr or Ag, to increase the electrical conductivity of these amorphous carbon coatings [10,11,12].

In addition to the enhancement of the electrical conductivity values, the use of silver as a doping element can also improve their corrosion resistance [10,13,14]. Yan [15] deposited carbon-like graphite (GLC) and titanium nitride (TiN) films with different Ag dopants on 316L stainless steel and Al substrate, and the coating showed excellent electrical conductivity. In the silver-doped conductive-corrosion-resistant coating, it is mainly dispersed in the form of nanocrystalized metal clusters [16]. Therefore, as a doping element, Ag will not significantly change the original bonding mode of the components in the coating, and Ag atoms will fill in a certain lattice position to form a typical nanocrymotic structure. However, the observed spontaneous escape behavior of silver limits its potential application in GLC coatings [10,17,18,19,20]. In previous studies, it was found that silver clusters separated at the interface of carbon clusters would escape under the influx of gas [21]. Studying how to stabilize silver in amorphous carbon films has important scientific significance and engineering value.

Silver surface segregation and clustering has also been observed in the development of several functional coatings, such as diamond-like carbon (DLC) [20,22], TiCN [23] or TiC/a-C:H [24] coatings. An important objective of these works was to understand how the silver distribution within the coating affects its functional properties. These analyses cover different aspects, such as mechanical properties (hardness or wear resistance), electrical properties, corrosion resistance or biological applications.

In this work, a series of the Ag-GLC coatings were prepared by the magnetron sputtering using a different silver target current and deposition temperature as well as the addition of CH_4_ gas flow. The main purpose of this work was to explore the influence of deposition parameters during magnetron sputtering on the silver’s spontaneous escape locations, the density of silver particles at the sample surface, the silver residue content and the corrosion performance of Ag-GLC coatings.

## 2. Experimental Procedures

### 2.1. Coatings Deposition Conditions

The silver-doped GLC coatings were prepared on the polished 6061 aluminum alloys and single-crystal silicon substrates using a MSIP 019 magnetron sputtering ion-plating system. For complete details on the preparation of targets, please refer to our previous work [21]. Firstly, the substrates were cleaned with dc sputtering for 20 min (current of chromium target (I_Cr_) 0.30 A, current of silver target (I_Ag_) 0.10 A, and a bias voltage of −400 V). Secondly, a pure Cr layer with thickness ca. 150 nm was deposited for 3 min (I_Cr_ 2.00 A and bias voltage of −120 V). Subsequently, three groups of samples were prepared with the current of both graphite targets (I_c_) at 1.50 A and the bias voltage at −60 V for 150 min. The other processing conditions were fixed as follows: (1) I_Ag_ = 0.01 A, 0.02 A, 0.03 A, and 0.05 A, deposition temperature 300 °C; (2) I_Ag_ = 0.03 A, deposition temperatures 200 °C, 400 °C and 500 °C; (3) I_Ag_ = 0.03 A, and deposition temperature 500 °C with CH_4_ gas flow at a rate of 20 mL/min. During the deposition process, the substrate holder was designed to separate the substrate form the target plate (ds-t) and fix it at 120 mm at a speed of 4 rev/min, while the thermocouple was also fixed at 120 mm without rotation. After the ion plating process, in order to explore the escape behaviour of silver, the specimens were exposed to the atmospheric environment for 3600 h.

### 2.2. Microscopic Characterization

The surface and cross-section morphologies of the silver-doped GLC coatings on single-crystal silicon substrates were characterized by field emission scanning electron microscopy (SEM, Carl Zeiss MERLIN), when exposed to the atmospheric environment (1 atm) for 3600 h. The content of the residual silver in the GLC coating was determined by an energy dispersive X-ray spectroscopy (EDS). Image Pro Plus 6.0 software (Media Cybernetics, Bethesda, MD, USA) was used to measure the density and average particle size of silver particles on the surface of the GLC coating.

A CH Instrument CHI 760D electrochemical analyzer and a conventional three-electrode cell were used to conduct the electrochemical tests in 0.5 mol/L H_2_SO_4_ solution containing 5 ppm F^−^ at 80 °C and bubbled O_2_ (cathode simulated). A platinum sheet with a surface area of 4 cm^2^ was used as counter electrode, while a saturated calomel electrode (SCE) was used as reference electrode. The working electrode (6061 Al alloy coated with Ag-GLC) was sealed with epoxy resin with an exposed surface area of 1 cm^2^. The potentiodynamic polarization curve was recorded in the range from −0.2 V vs. open-circuit voltage to +1.0 V vs. SCE at a potential scanning rate of 1 mV/s after immersion for 0.5 h in the electrolyte solution.

## 3. Result and Discussion

### 3.1. The Infiuence of the Silver Target Current (I_Ag_) on the Spontaneous Escape Behavior of Silver

**Figure 1:**
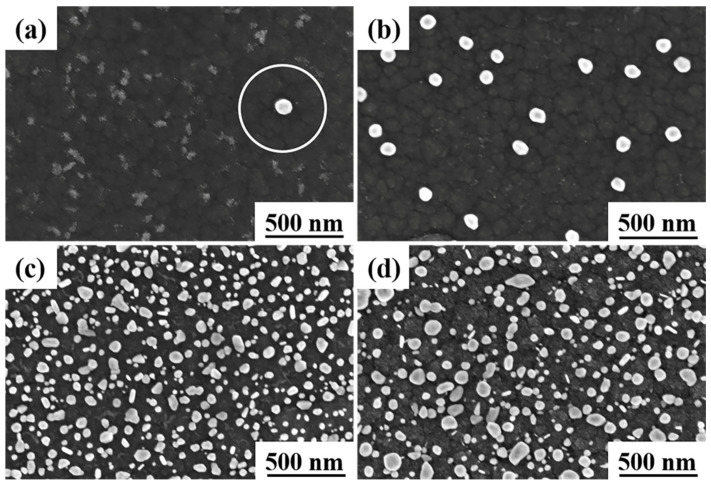
After 3600 h exposure in an atmospheric environment, surface morphologies of the Ag-GLC coatings prepared at 300 °C with different I_Ag_: (**a**) I_Ag_ = 0.01 A, (**b**) I_Ag_ = 0.02 A, (**c**) I_Ag_ = 0.03 A, and (**d**) I_Ag_ = 0.05 A.

Figure 1 shows the surface morphology of Ag-GLC coatings prepared with different I_Ag_ at 300 °C after being exposed to air (1 atm) for 3600 h. It can be seen that the spontaneous escape phenomenon of silver could take place, regardless of I_Ag_. The escaped silver clusters or particles did not cover the whole surface of the GLC coating. However, the value of I_Ag_ had a great impact on the distribution of the escaped silver particles on the sample surface. Table 1 shows the coverage of the escaped silver particles on the coating surface.

When the Ag-GLC coatings were prepared with I_Ag_ = 0.01 A, a bimodal particle distribution was found. Most of the silver particles were smaller than 10 nm, which tended to agglomerate in clusters and were located very close to the three-point grain boundaries. Moreover, as indicated by the circle in Figure 1a, around the big silver particle, the amount of silver clusters was strongly reduced in comparison with the rest of the surface. This can also explain the phenomenon that, in the case of I_Ag_ = 0.02 A (Figure 1b), the bigger particles were predominant with a small number of silver clusters. On the contrary, when the I_Ag_ increased to 0.03 A and 0.05 A, the samples exhibited a completely different Ag particle distribution (Figure 1c,d). The escaped silver particles can be classified in three different size ranges, and their fraction of surface coverage was significantly improved (Table 1).

**Figure 2:**
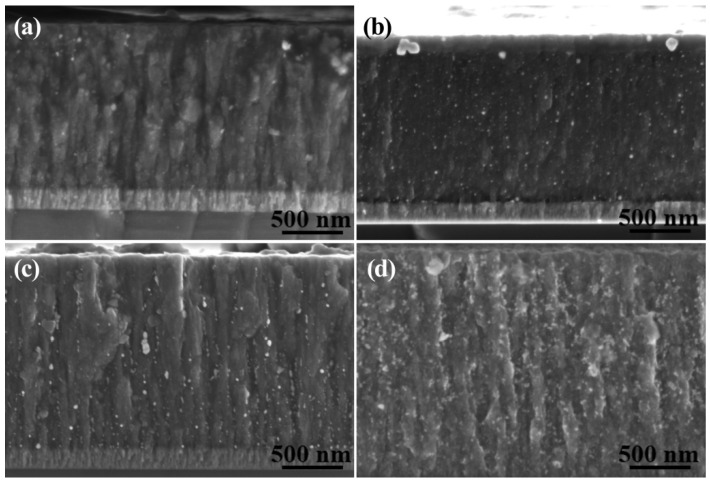
After 3600 h exposure in an atmospheric environment, cross-sectional morphologies of the Ag-GLC coatings prepared at 300 °C with different I_Ag_: (**a**) I_Ag_ = 0.01 A, (**b**) I_Ag_ = 0.02 A, (**c**) I_Ag_ = 0.03 A, and (**d**) I_Ag_ = 0.05 A.

Figure 2 reveals the cross-section morphologies of the Ag-GLC coatings prepared with different I_Ag_. The thickness of the GLC coatings increased by 0.3 μm along with the increase in I_Ag_ from 0.01 A up to 0.05 A. The silver residue content inside the GLC coatings is given in Table 1. It increased with the I_Ag_, and reached a maximum value of 9.72% when the applied I_Ag_ was 0.05 A. The silver volume percentage in the GLC coating could be approximately estimated by adding the content of silver remaining in the coating and the silver escaping to the surface. It increased from 3.5 % (I_Ag_ = 0.01 A) to 17.2 % (I_Ag_ = 0.05 A). This is reflected in the increase in the coating thickness that was measured in the different samples (Table 2); this was 22% higher in the sample processed with I_Ag_ = 0.05 A in comparison with the first sample, processed with I_Ag_ = 0.01 A. The fact that the increase in coating thickness was higher than the silver volume percentage introduced during the deposition process suggested that the connectivity of the carbon network could be deteriorated when a higher I_Ag_ value was used.

**Figure 3:**
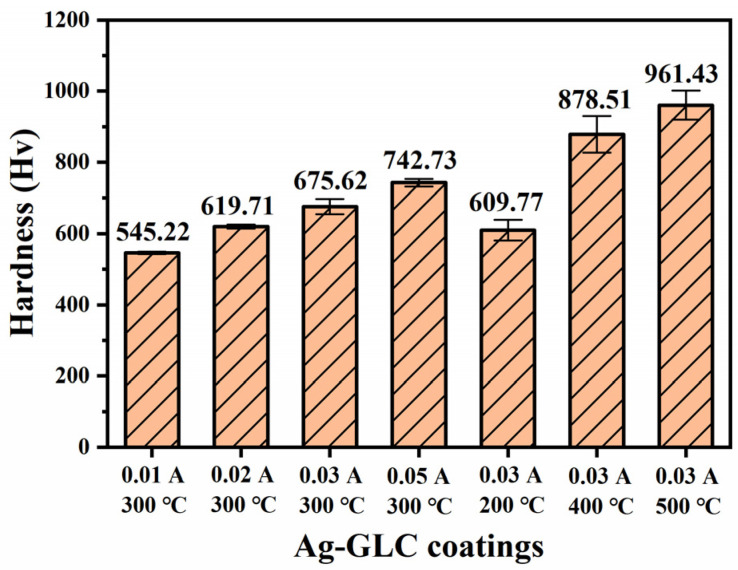
The hardness of the Ag-GLC coatings prepared at different deposition parameters.

Figure 3 exhibits the hardness of the Ag-GLC coatings prepared at different deposition parameters. It can be seen that both silver target current and deposition temperature affected the hardness of the Ag-GLC coating, which increased with the increase in the silver target current and the deposition temperature. The maximum hardness value was Hv 961.43 when IAg was 0.03 A and deposition temperature was 500 °C.

### 3.2. The Influence of Deposition Temperature on the Spontaneous Escape Behavior of Silver

**Figure 4:**
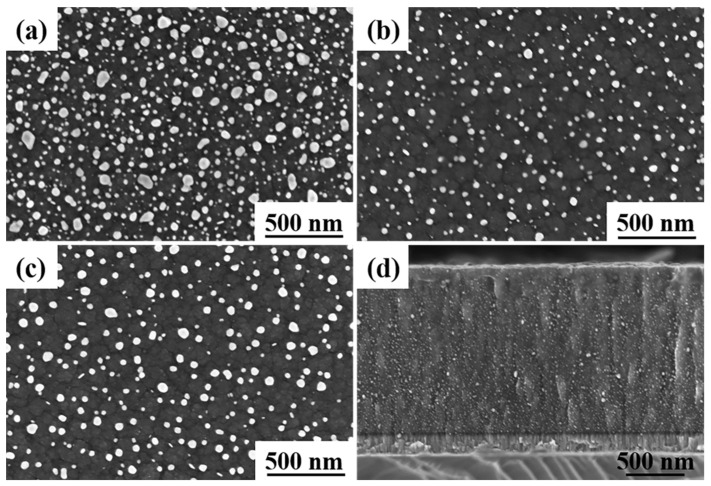
After 3600 h exposure in an atmospheric environment, surface morphologies of the Ag-GLC coatings prepared with I_Ag_ = 0.03 A at different deposition temperature: (**a**) 200 °C, (**b**) 400 °C, (**c**) 500 °C, and (**d**) cross-sectional morphologies of the Ag-GLC coating prepared at 500 °C.

Figure 4 gives the surface morphologies of the Ag-GLC coatings obtained at 0.03 A with different deposition temperatures. Taking the Ag-GLC coatings prepared at 300 °C into account (Figure 1c), two different silver particle distribution configurations are also detected. In the samples treated at the lower temperatures (200 °C and 300 °C), the fractions of surface coverage of the escaped silver particles were above 20% (Table 1). By contrast, when the deposition temperatures reached 400 °C and 500 °C, the escaped silver particles were refined to some extent, and their numbers were visibly decreased (Figure 4b,c). Thus, the surface coverage fractions of the escaped silver particles fell to below 10% (Table 1).

Since the cross-section morphologies of the Ag-GLC coatings obtained with different deposition temperatures were very similar, only the one produced at 500 °C is presented in Figure 4d. Fine silver particles can be observed to be more uniformly distributed in the GLC coating, compared with that obtained at 300 °C, in Figure 2c. EDS elemental mapping was adopted to determine the silver residue content inside the GLC coating. A small increase in the silver residue content was measured when the deposition temperature increased: 5.11 at.% (200 °C), 5.12 at.% (300 °C), 5.52 at.% (400 °C), and 5.58 at.% (500 °C). It has been testified that the compact carbon cluster interfaces are easier to form at higher deposition temperature [25,26], which could effectively hinder the escape process of the large silver particles from the GLC coatings. Hence, with elevated deposition temperature, the GLC coating possesses a relatively high silver residue content, and fewer silver particles can escape to the coating surface.

### 3.3. Effect of CH_4_ Gas Flow on the Spontaneous Escape Behavior of Silver

**Figure 5:**
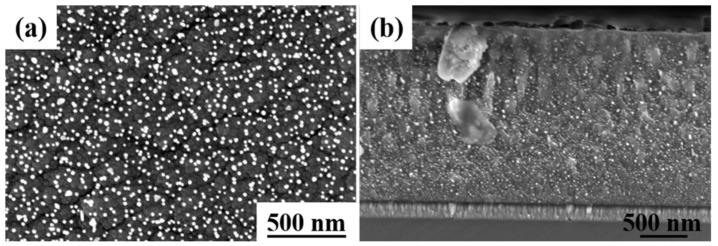
After 3600 h exposure in an atmospheric environment, surface (**a**) and cross-sectional (**b**) morphologies of the Ag-GLC coatings prepared at 500 °C with I_Ag_ = 0.03 A and CH_4_ gas flow of 20 sccm.

The last sample was prepared at 500 °C with I_Ag_ = 0.03 A and CH_4_ gas flow of 20 mL/min, and its microstructure after exposure to atmospheric pressure for 3600 h is shown in Figure 5. The escaped silver particles with mean diameters in the range of 20–35 nm were distributed uniformly on the surface of the GLC coating (Figure 5a). Most of the escaped silver particles existed in the interior of the amorphous carbon clusters, while few silver particles were located on the interface of the amorphous carbon clusters. In addition, these silver particles tended be ordered following a linear structure, with sets of three to six particles perfectly ordered. The cross-section of this sample (Figure 5b) also exhibited several differences in comparison with the samples processed in argon. The thickness was about 2.5 μm, 800 nm larger than the other Ag-GLC coatings above. Finally, it is worth noting that, from the macroscopic surface, the Ag-GLC coatings formed with CH_4_ gas flow were found to have peeled off in some areas, indicating a poor adhesion with the substrate.

### 3.4. Discussion of the Spontaneous Escape Behavior of Silver from the GLC Coatings

**Figure 6:**
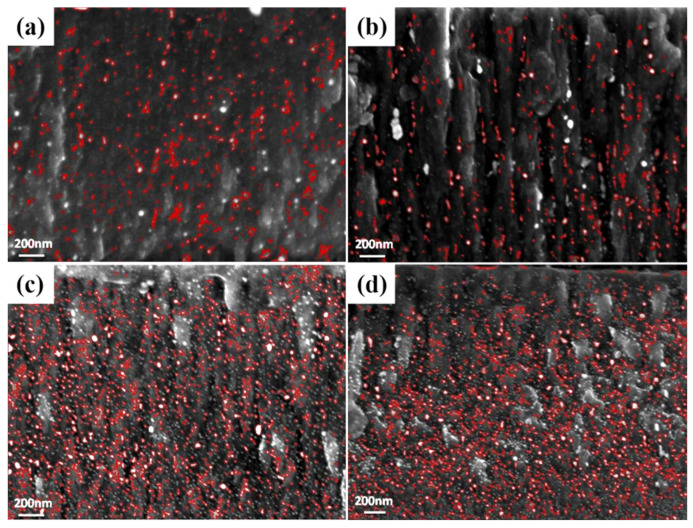
After 3600 h exposure in an atmospheric environment, the silver distribution in the cross-section of the Ag-GLC coatings prepared at different deposition parameters: (**a**) 0.02 A, 300 °C, (**b**) 0.03 A, 300 °C, (**c**) 0.03 A, 500 °C and (**d**) 0.03 A, 500 °C and CH_4_ gas.

In order to explain the differences observed in the above sections, the distribution of silver particles in the cross-section of the GLC coatings was analyzed in detail. Figure 6 reveals the cross-section of the several samples, highlighting the position and size of the silver particles in order to increase the contrast. In all the cases, it is clear that there exists a gradient in silver composition along the direction of the coating cross-section. Meanwhile, the silver particles which migrate to the surface mainly come from the outer part of the coating, and the size of these silver particles is very similar, with a near-Gaussian distribution in average sizes of 16–19 nm. The GLC coatings exhibited a structure resembling the columnar structure of the bottom Cr layer, likely a consequence of the shadow effect present during the coating deposition.

These phenomena in Figure 6 are consistent with our previous study [21]. Our previous research confirmed that, when the Ag-GLC coatings are moved from a vacuum chamber (10^−4^ Torr) into atmosphere (1 atm), under the huge pressure difference the gas molecules in air can easily rush into and fill up the carbon cluster interface, and the Ag atoms and clusters distributed on the outer layer of the GLC coating will escape in the case of gas influx [21]. Afterwards, the escaped silver atoms or clusters spread along the surface to form large silver clusters or particles. This process is driven spontaneously by the reduction in surface free energy [21]. Wettergren et al. [27] and Zhdanov et al. [28] have reported a similar particle growth process described as Ostwald ripening. The clusters or very small nanoparticles tend to adhere onto the larger nanoparticles, and the latter grow in size at the expense of the former [27,28].

**Figure 7:**
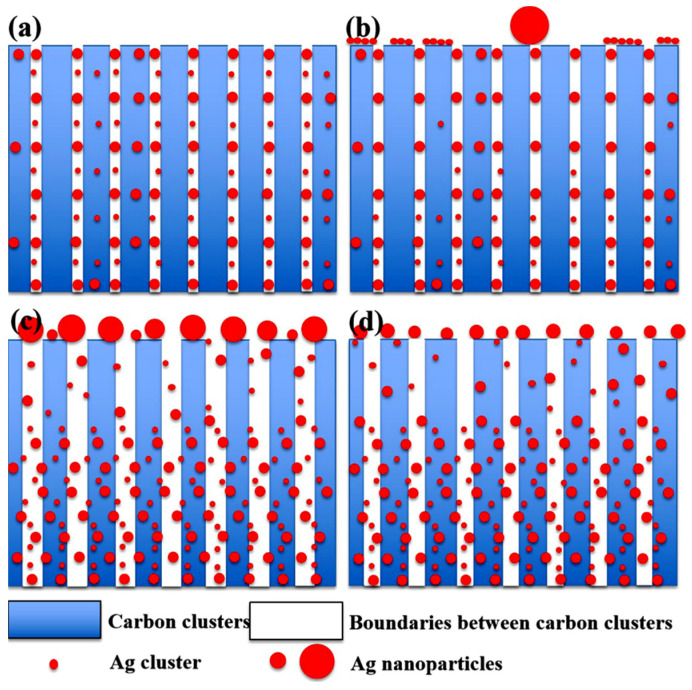
Schematic diagrams for the spontaneous escape behavior of silver from the GLC coatings: (**a**) the original silver distribution in the Ag-GLC coating; (**b**) in the case of the GLC coatings fabricated with low I_Ag_ (0.01 A and 0.02 A at 300 °C), the escaped silver clusters grow in size by absorbing the surrounding little ones; (**c**) the silver content inside the Ag-GLC coatings increased by using the high I_Ag_ (0.03 A and 0.05 A at 300 °C); and (**d**) with the augmentation of deposition temperature (400 °C and 500 °C with I_Ag_ = 0.03 A), only a small number of silver particles of small size are able to escape from the GLC coatings.

According to the discussion above, the spontaneous escape behavior of the silver from the GLC coatings can be elucidated in Figure 7. In the case of the GLC coatings fabricated with low I_Ag_ (0.01 A and 0.02 A at 300 °C) (Figure 7b), the number of the dispersed silver particles is very low, and thus they do not disturb the packaging of the carbon grains. The GLC coating will block the movement of the big silver clusters and only the small ones can migrate to the surface. Once they reach the surface, large silver clusters or particles can be formed due to the reduction in the surface free energy (Figure 7b).

When the Ag target current increases, the amount of silver particles between the carbon grains increases, resulting in a reduction in the C–C grain connectivity. In this situation, it is not only the smaller silver particles that can migrate, but also the bigger ones. These big silver particles have a lower mobility on the surface and, for this reason, shorter distances between the silver particles in the coating surface were measured and a broader size distribution was observed (Figure 7c).

During the magnetron sputtering process, the sputtered ion energy was proportional to the deposition temperature, and the higher deposition temperature was conducive to improving the interdiffusion of atoms in the Ag-GLC coatings. Therefore, studies have proven that the more compact carbon cluster interface could be obtained at a higher substrate temperature [25,26]. With the augmentation of deposition temperature, more silver particles were trapped in the GLC coating (Table 2), and only a small number of silver particles of small size were able to escape from the GLC coatings (Figure 4).

### 3.5. Electrochemical Corrosion of the Ag-GLC Coatings

**Figure 8:**
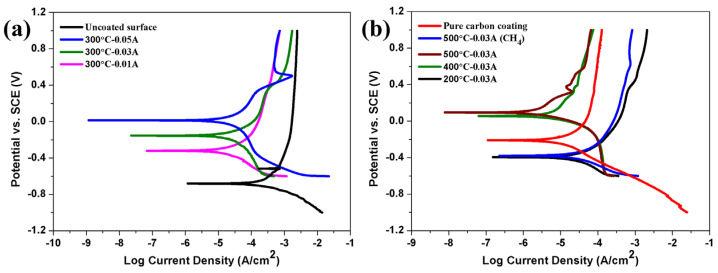
Potentiodynamic polarization curves of the Ag-GLC coatings in 0.5 mol/L H_2_SO_4_ + 5 ppm F^-^ solution at 80 °C, purged with air: (**a**) prepared at 300 °C with different I_Ag_ and (**b**) prepared with I_Ag_ = 0.03 A at different deposition temperature and with the pure carbon coating.

The corrosion resistances of the different Ag-GLC coatings were evaluated using potentiodynamic polarization, and the results are presented in Figure 8 The corrosion potential (*E_corr_*) and corrosion current density (*I_corr_*) derived from the Tafel curves are listed in Table 3. Based on the results in Figure 8a and Table 3, it can be seen that the GLC and Ag-GLC coatings helped to greatly enhance the anti-corrosion properties of the 6061 Al alloy. However, at the deposition temperature of 300 °C, the addition of silver decreased the corrosion resistance of the GLC coating. In addition, the rise in I_Ag_ was beneficial to improve the *E_corr_* of the Ag-GLC coatings, but had little effect on their *I_corr_*. The silver residue content inside the GLC coating increased with I_Ag_, and thus lowered the connectivity of the GLC coating. Therefore, changing the I_Ag_ value had little effect on enhancing the corrosion resistance of the Ag-GLC coatings.

**Figure 9:**
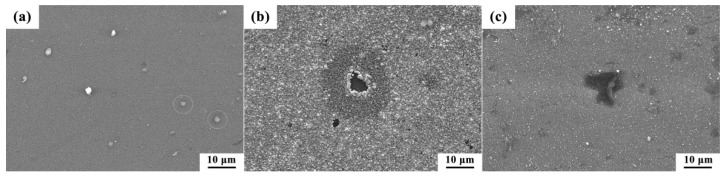
Surface morphology of Ag-GLC coatings after corrosion: (**a**) 0.03 A, 500 °C, (**b**) 0.05 A, 300 °C, and (**c**) 0.03 A, 300 °C.

By analyzing the results in Figure 8b and Table 3, it was concluded that the corrosion resistance of the Ag-GLC coatings was effectively enhanced by the changing deposition temperature for the increasing *E_corr_* and decreasing *I_corr_*. In particular, when the substrate temperature rose to 400 °C and 500 °C, the Ag-GLC coatings exhibited a better corrosion resistance than the GLC coating, which was closely related to the lower defect density of the GLC coatings prepared at a high temperature [26]. Moreover, it is worth noting that adding CH_4_ gas flow during the deposition was detrimental to the corrosion resistance of the Ag-GLC coatings. This was largely due to the fact that the addition of CH_4_ gas flow destroyed the adherence of the GLC coating to the substrate. According to the polarization studies combined with the SEM images, the GLC coating prepared at 500 °C with I_Ag_ = 0.03 A displayed the best corrosion resistance compared with the other specimens, as shown by the lowest corrosion current density and relatively compact uniform microstructure. Figure 9 shows the surface morphology of Ag-GLC coatings after corrosion. It can be seen that there were slight corrosion spots and bulges on the coating surface (Figure 9a,c). It is worth noting that large corrosion pits appeared in the Ag-GLC coating (Figure 9b). This also proved that the Ag-GLC coating prepared at 500 °C was the most resistant to corrosion. Thus, the material prepared with these conditions appears to be more suitable for PEMFC environments.

## 4. Conclusions

This work has primarily investigated the effects of the deposition parameters on the spontaneous escape behavior of silver from the GLC coatings prepared by magnetron sputtering. The main conclusions that can be drawn are as follows:

1. The silver target current directly decided the silver content incorporated into the GLC coating and increased the coating thickness, but it played a minor role in enhancing the corrosion resistance of the GLC coatings.
2. When the silver target current was fixed, the deposition temperature did not change the initial content of silver particles incorporated into the GLC coatings. However, increasing the deposition temperature effectively reduced the number of silver particles escaping from the GLC coatings. Meanwhile, as it rose up to 400 °C and 500 °C, the corrosion resistance of the GLC coating was clearly enhanced.
3. Compared to the Ag-GLC coatings prepared by argon under the same conditions, after using CH_4_ gas flow, the escaped silver particles were obviously refined, but these Ag-GLC coatings had a poor adhesion with the substrate. Hence, the use of CH_4_ gas flow actually led to a worsening of the corrosion resistance of the coating.

## Figures and Tables

**Table 1:** The size distribution of silver particles on the surface of the Ag-GLC coatings formed at different deposition parameters.

Deposition Parameters (T, °C; I_Ag_, A)	300 0.01	300 0.02	300 0.03	300 0.05	200 0.03	400 0.03	500 0.03
Area covered with Ag (%)	3.2	5.0	27.0	25.1	20.3	8.0	10.0

**Table 2:** The thickness of the Ag-GLC coatings and the silver content in the Ag-GLC coatings prepared at different deposition parameters.

Temperature (°C) I_Ag_ (A)	300 0.01	300 0.02	300 0.03	300 0.05	200 0.03	400 0.03	500 0.03
Coating thickness (μm)	1.50	1.54	1.70	1.84	1.64	1.65	1.52
Initial Ag content (vol.%)	3.5	5.2	9.5	17.2	9.5	9.5	9.5
Ag residue content (at.%)	2.31	3.35	5.12	9.72	5.11	5.52	5.58

**Table 3:** Fitting results of potentiodynamic polarization tests.

Samples	*E_corr_* (mV) (SCE)	*I_corr_* (μA cm^−2^)
Uncoated 6061	−681	296.90
Pure carbon coating	−21	12.55
0.01 A—300 °C	−320	18.81
0.03 A—300 °C	−154	27.62
0.05 A—300 °C	14	23.89
0.03 A—200 °C	−395	30.95
0.03 A—300 °C	−154	27.62
0.03 A—400 °C	56	7.69
0.03 A—500 °C	95	3.16
0.03 A—500 °C (CH_4_)	−379	30.86

## Data Availability

Not applicable.
